# Depression, anxiety, and stress among Iranian nurses in COVID-19 care wards

**DOI:** 10.1186/s40359-022-00911-8

**Published:** 2022-08-20

**Authors:** Azam Sharifi, Masoud Fallahi-Khoshknab, Shamaneh Mohammadi, Mashaallah Zeraati, Zahra Jamshidi, Mohsen Aghabeygi-Arani, Nilofar Mirzaei, Negin Fallahi-Khoshknab, Parisa Rasooli

**Affiliations:** 1 Nursing Department, Nahavand School of Allied Medical Sciences, Hamadan University of Medical Sciences, Hamadan, Iran; 2 Nursing Department, University of Social Welfare and Rehabilitation Sciences, Tehran, Iran; 3 Health System Research Committee, Shiraz University of Medical Sciences, Shiraz, Iran; 4 Shahid Rahimi Hospital in Khorramabad, Lorestan University of Medical Sciences, Khorramabad, Iran; 5 Medical Student, Faculty of Medicine, Alborz University of Medical Sciences, Karaj, Iran

**Keywords:** Coronavirus disease 2019, Stress, Anxiety, Depression, Nurse

## Abstract

**Background:**

Nurses are at the frontline of care provision to patients with coronavirus disease 2019 (COVID-19). The high communicability of COVID-19, high levels of stress associated with the disease, and challenges of care provision to afflicted patients faced nurses in Iran with problems such as depression, anxiety, and stress. The aim of the study was to assess depression, anxiety, and stress among Iranian nurses who provided care to patients with COVID-19.

**Methods:**

This cross-sectional descriptive-analytical study was conducted in 2020–2021. Participants were 468 nurses purposively selected from university hospitals in Iran. They completed two online instruments, namely a demographic questionnaire and the 21-item Depression Anxiety Stress Scale. Data were analyzed using the SPSS software (v. 23.0).

**Results:**

Most participants were female (75.9%) and married (73.4%) and held bachelor’s degree (88%). The means of participants’ age and work experience were 33.59 ± 6.40 years and 10.26 ± 6.61 years, respectively. The mean scores and the prevalence rates of depression, anxiety, and stress were 13.56 ± 5.37 and 74.1%, 13.21 ± 4.90 and 89.7%, and 15.13 ± 4.76 and 54.9%, respectively. The prevalence rates of moderate to severe depression, anxiety, and stress were 43.7%, 73%, and 24%, respectively. The mean scores of participants’ depression, anxiety, and stress had significant relationship with their employment status (*p* < 0.05). Besides, the mean scores of their anxiety had significant relationship with their educational level, employment status, and work shift (*p* < 0.05).

**Conclusion:**

Most nurses who provide care to patients with COVID-19 suffer from depression, anxiety, and stress. Psychological support services may be needed for nurses in order to protect and promote their mental health.

## Background

Coronavirus disease 2019 (COVID-19) is an emerging infectious disease first isolated and reported in January 7, 2020, in Wuhan, China, and rapidly spread worldwide [1]. By November 3, 2021, the total number of afflicted patients and the total number of deaths due to COVID-19 in the world were 248,385,611 and 5,031,006, respectively [2]. COVID-19 can be transmitted through close contacts and has an average incubation period of five days, which may sometimes reach to fourteen days. Almost all afflicted patients experience one or more symptoms during the first 5–12 days after affliction. The symptoms of COVID-19 widely vary so that some patients may be asymptomatic or experience the mild symptoms of upper respiratory tract infection, while some patients may experience severe respiratory symptoms. Fever is often the most common symptom which may appear alone or in association with dry cough, dyspnea, myalgia, dizziness, headache, sore throat, rhinorrhea, chest pain, nausea, and vomiting. Studies show that acute respiratory distress syndrome happens in almost 15% of afflicted patients and 50%–85% of afflicted patients hospitalized in intensive care unit experience hypoxia and respiratory distress [3–5].

Nurses, as the largest group of healthcare providers, have significant role in care provision to healthcare clients [6]. Nursing is a highly stressful job and nurses usually have high levels of physical and mental workload [7]. During epidemics, nurses face serious health threats due to the necessity of using heavy personal protective equipment, risk of affliction by infection, and risk of infection transmission to others [8, 9]. Nurses are at the frontline of care provision to COVID-19-afflicted patients and spend a great deal of time in close contact with them and hence, are greatly at risk for affliction by the disease or transmission of the disease to others [10, 11]. On the other hand, the COVID-19 pandemic has increased nurses’ workload due to the high levels of stress associated with the pandemic, frequent changes in the behaviors of the virus, and high communicability of the disease, and led to the shortage of equipment, physical burnout, and ethical distress for nurses [12–15]. Besides, nurses in Iran face problems such as inappropriate work schedule, inadequate organizational support, shortage of experienced staff, and limited specialized education about COVID-19 [16]. The healthcare system of Iran also suffers from serious problems such as lack of medical equipment, lack of COVID-19 diagnostic kits, and lack of COVID-19 vaccines due to international sanctions. On the other hand, factors such as limited public adherence to COVID-19 guidelines, limited public trust in media, large number of individuals who request healthcare services in healthcare settings, and poor healthcare management and planning have significantly increased the prevalence and mortality rates of COVID-19 in Iran [17, 18]. These problems have caused high levels of stress and anxiety for nurses [10, 16].

Stress is an actual or interpreted threat to physiological or psychological integrity which leads to physiological or behavioral responses [19]. Occupational stress is an interaction between occupational conditions and workers which is associated with changes in employee’s physiological and psychological status and functioning [20, 21]. Anxiety is also defined as the prediction of a threat in future and is characterized by disturbing feelings such as uncertainty, horror, and fear [22, 23]. Long-term stress and anxiety can lead to depression which is a prevalent and serious medication condition with negative effects on feelings, thinking, and functioning. Depression can in turn lead to sorrow, unhappiness, loss of interest in previously enjoyable activities, different physical and emotional problems, and reduced functional ability [24].

Studies showed that during the Severe Acute Respiratory Syndrome epidemic in 2003 in Taiwan and Singapore, nurses who provided care to afflicted patients suffered from mental health problems such as anxiety, depression, and hostility [25–27]. Similarly, nurses who provided care to patients with Middle East Respiratory Syndrome reported problems such as fear, anger, and mental distress [28]. The results of a study also showed that the prevalence of mental health problems among nurses who provided care to patients with COVID-19 was 32.9% for posttraumatic stress disorder, 75.3% for anxiety, and 28.8% for depression [10]. Another study in China showed that more than 70% of healthcare providers who provided care to patients with COVID-19 suffered from mental health problems such as anxiety, depression, and sleeplessness [29]. The negative effects of infectious disease epidemics on mental health among healthcare providers can last for long times after the end of the epidemics and may lead to problems such as depression, stress, and posttraumatic stress disorder [10, 13, 14].

Previous studies reported the significant effects of mental disorders among nurses on the quality of their care services [15, 30]. Therefore, careful attention to nurses’ mental health is needed. Given the significant effects of the COVID-19 pandemic on nurses’ mental health, context-based studies in different areas are needed to assess nurses’ psychological experiences of care provision to afflicted patients. Similarly, regular screening for their mental health problems, particularly stress, anxiety, and depression, is essential [31, 32]. Nonetheless, there are limited data in this area in Iran and hence, the present study was conducted to reduce this gap and help policy makers develop culturally appropriate managerial and protective plans. The aim of the study was to assess depression, anxiety, and stress among Iranian nurses who provided care to patients with COVID-19. We had the hypothesis that depression, anxiety, and stress were highly prevalent among Iranian nurses who provide care to patients with COVID-19.

## Methods

### Study design

This cross-sectional descriptive-analytical study was conducted from May 2020 to March 2021 based on the Strengthening the Reporting of Observational studies in Epidemiology (STROBE) guideline [33].

### Participants and setting

The statistical population of the study consisted of nurses in the COVID-19 care wards of hospitals affiliated to medical sciences universities in Iran. Using the Cochran formula and with a confidence level of 0.95, a power of 0.90, and an effect size of 0.05, sample size was calculated to be 384 [34]. Sampling was purposively performed based on the following criteria: care provision to patients with COVID-19 in COVID-19 care wards, work experience of at least one month in COVID-19 care wards, work experience of at least one year in nursing, no significant life events (such as significant losses, divorce, etc.) in the past six months, no self-reported use of antidepressant, anxiolytic, or psychotropic agents, and agreement for participation. Participants who incompletely answered the study instruments were excluded.

### Instruments

Instruments for data collection were demographic and occupational characteristics information and the Depression Anxiety Stress Scale (DASS) [35]. The demographic and occupational characteristics information had nine items on age, gender, marital status, educational level, work experience, affiliated COVID-19 ward (emergency ward, intensive care unit, or internal medicine ward), employment status, work shift, and number of overtime work hours per month.

The original Depression Anxiety Stress Scale (DASS) has 42 items in the three subscales of depression, anxiety, and stress. Its short form, used in the present study, has 21 items in three seven-item subscales. This scale is a standard self-report instrument developed by Antnoy et al. in 1988 for assessing the symptoms of depression, anxiety, and stress among individuals without the diagnosis of depression, anxiety, and stress [35, 36]. Items are scored on a four-point scale from zero (“Does not apply to me at all”) to 3 (“Applies to me very much”). Therefore, the possible total score of each seven-item subscale can be 0–21. As the 21-item scale is the short form of the 42-item scale, its scores should be doubled for interpretation as normal, mild, moderate, severe, and extremely severe as shown in Table 1 [35–38]. The total score is not reported for this scale. Previous studies confirmed the acceptable validity and reliability of this scale [39–42]. A study in Iran assessed the psychometric properties of this scale and reported that its Cronbach’s alpha and test–retest correlation coefficients were 0.7 and 0.81 for its depression subscale, 0.67 and 0.73 for its anxiety subscale, and 0.49 and 0.81 for its stress subscale [39].

**Table 1:** Interpretation of the scores of the DASS subscales

Subscales	Interpretation				
	Normal	Mild	Moderate	Severe	Extremely severe
Depression	0–9	10–13	14–20	21–27	28 +
Anxiety	0–7	8–9	10–14	15–19	20 +
Stress	0–14	15–18	19–25	26–33	34 +

### Data collection

Data collection instruments were distributed among nurses online in the study setting through WhatsApp, Telegram, or Eta applications and they were asked to complete them. Nurses who completed and sent us the instruments were assessed for eligibility and were included in the study if satisfied the eligibility criteria.

### Data analysis

Data were analyzed using the SPSS software (v. 23.0). Data normality was tested through the Shapiro–Wilk test which showed the normal distribution of the mean scores of depression (*p* = 0.126), anxiety (*p* = 0.175), and stress (*p* = 0.252). The measures of descriptive statistics (namely frequency distribution, mean, and standard deviation) were used for data summarization and description. The Pearson’s correlation analysis was used to assess the correlation of the mean scores of depression, anxiety, and stress with age, work experience, and overtime work per month. Moreover, the differences in the mean scores of depression, anxiety, and stress with categorical variables including gender, marital status, educational level, affiliated ward, and employment status were tested through the independent-sample *t* test and the one-way analysis of variance. The multiple linear regression analysis with the Stepwise method was also used to determine the predictors of depression, anxiety, and stress. Independent variables were variables that had significant relationship with depression, anxiety, and stress and were separately entered into the model. The level of significance was set at less than 0.05.

### Ethical considerations

The Ethics Committee of the University of Social Welfare and Rehabilitation Sciences, Tehran, Iran, approved this study (code: IR.USWR.REC.1399.105). Participants completed and signed the online informed consent form of the study which included information about the authors, aim of the study, confidentiality of the study data, and voluntariness of participation in and withdrawal from the study. They could access and complete the study instruments only if they signed the informed consent form of the study.

## Results

A total of 479 nurses from COVID-19 care wards answered the study instruments. Eleven nurses (2.3%) incompletely answered the instruments and were excluded and final data analysis was performed on the data obtained from 468 nurses. Most participants were female (75.9%) and married (73.4%), held bachelor’s degree (88%), worked rotating shifts (72.1%), and had official employment (51.3%). The means of participants’ age, work experience, and overtime work per month were 33.59 ± 6.40 years, 10.26 ± 6.61 years, and 59.06 ± 36.04 h, respectively (Table 2).

**Table 2:** Participants’ demographic characteristics and their relationships with the mean scores of depression, anxiety, and stress^*^

Characteristics		N (%) or Mean ± SD	Stress Mean ± SD	Anxiety Mean ± SD	Depression Mean ± SD
Gender	Female	355 (75.9)	15.23 ± 4.73	13.16 ± 4.92	13.56 ± 5.48
	Male	113 (24.1)	14.87 ± 4.89	13.34 ± 4.86	13.58 ± 5.10
	Test results^a^		*t* = 0.706 *p* = 0.553	*t* = 0.337 *p* = 0.453	*t* = 0.038 *p* = 0.414
Age (Years)	–	33.59 ± 6.40	15.13 ± 4.76	13.21 ± 4.90	13.56 ± 5.37
	Test results^b^		r = 0.02 *p* = 0.634	r = 0.03 *p* = 0.423	r = 0.01 *p* = 0.865
Marital status	Married	333 (71.2)	15.06 ± 4.70	13.26 ± 5.05	13.51 ± 5.55
	Single	135 (28.8)	15.30 ± 4.90	13.09 ± 4.54	13.70 ± 4.95
	Test results^a^		*t* = 0.501 *p* = 0.355	*t* = 0.359 *p* = 0.073	*t* = 0/353 *p* = 0.503
Educational level	Bachelor’s	412 (88.0)	15.07 ± 4.70	13.25 ± 4.76	13.55 ± 5.37
	Master’s	56 (12.0)	15.59 ± 5.24	12.94 ± 5.85	13.67 ± 5.42
	Test results^a^		*t* = 0.772 *p* = 0.415	*t* = 0.436 *p* = 0.013	*t* = − 0.153 *p* = 0.461
Affiliated COVID-19 care ward	Internal medicine	208 (44.4)	14.62 ± 4.52	13.00 ± 4.56	13.43 ± 5.88
	Intensive care	158 (33.8)	15.81 ± 4.91	13.63 ± 5.17	13.89 ± 5.19
	Emergency	102 (21.8)	15.09 ± 5.00	13.30 ± 4.91	13.84 ± 5.21
	Test results^c^		F = 1.884 *p* = 0.131	F = 0.916 *p* = 0.433	F = 0.886 *p* = 0.448
Work experience (Years)	–	6.61 ± 0.26	15.13 ± 4.76	13.21 ± 4.90	13.56 ± 5.37
	Test results^b^		r = 0.01 *p* = 0.927	r = 0.03 *p* = 0.478	r = 0.01 *p* = 0.824
Employment status	Permanent official	240 (51.3)	14.90 ± 4.58	13.02 ± 5.02	13.42 ± 5.48
	Conditional official	66(14.0)	14.61 ± 4.67	12.18 ± 4.25	12.48 ± 4.98
	Contractual	84 (17.9)	14.94 ± 4.91	13.61 ± 5.09	13.45 ± 5.75
	Mandatory post-graduation service	78 (16.7)	16.82 ± 5.55	15.05 ± 4.87	15.24 ± 5.17
	Test results^c^		F = 1223 *p* = 0.015	F = 3.462 *p* = 0.008	F = 2.996 *p* = 0.018
Overtime work per month (Hours)	–	59.05 ± 36.04	15.13 ± 4.76	13.21 ± 4.90	13.56 ± 5.37
	Test results^b^		r = 0.02 *p* = 0.586	r = 0.04 *p* = 0.389	r = 0.02 *p* = 0.549
Work shift	Day	79 (16.9)	14.75 ± 4.72	12.89 ± 4.90	13.29 ± 4.76
	Night	44 (9.4)	15.77 ± 5.07	15.23 ± 5.07	15.02 ± 5.30
	Rotating	345 (73.7)	15.14 ± 4.74	13.04 ± 4.83	13.45 ± 5.50
	Test results^c^		F = 0.655 *p* = 0.520	F = 4.009 *p* = 0.019	F = 1.684 *p* = 0.187

*Depression Anxiety Stress Scale (DASS-21)[35]

^a^The results of the independent-sample *t* test

^b^The results of the Pearson’s correlation analysis

^c^The results of the one-way analysis of variance

The mean scores of participants’ depression, anxiety, and stress were 13.56 ± 5.37, 13.21 ± 4.90, and 15.13 ± 4.76, respectively. Moreover, the overall prevalence rates of depression, anxiety, and stress were 74.1%, 89.7%, and 54.9% and the prevalence rates of moderate to severe stress, anxiety, and depression were 43.7%, 73%, and 24%, respectively (Table 3).

**Table 3:** The severity of participants’ depression, anxiety, and stress*

Severity	Problem		
	Depression	Anxiety	Stress
	N (%)	N (%)	N (%)
Normal	121 (25.9)	48 (10.3)	211 (45.1)
Mild	142 (30.4)	78 (16.7)	146 (31.2)
Moderate	156 (33.3)	182 (38.9)	99 (21.1)
Severe	39 (8.3)	105 (22.4)	12 (2.6)
Extremely severe	10 (2.1)	55 (11.7)	0 (0)
Mean ± SD	13.56 ± 5.37	13.21 ± 4.90	15.13 ± 4.76

*Depression Anxiety Stress Scale (DASS-21)[35]

Relationship analysis revealed that the mean scores of participants’ depression, anxiety, and stress had no significant relationship with their age, gender, affiliated ward, work experience, and overtime work hours per month. However, the mean score of their anxiety had significant differences with their educational level, employment status, and work shift and the mean scores of their depression and stress had significant differences with their employment status (*p* < 0.05). The results of the Tukey’s post hoc analysis showed that the mean scores of depression, anxiety, and stress among nurses who were doing their mandatory post-graduation services were significantly greater than all other nurses (*p* < 0.05). Moreover, nurses with master’s degree obtained significantly lower anxiety scores than nurses with bachelor’s degree and nurses with night work shift obtained significantly higher anxiety scores than nurses with day or rotating shift (*p* < 0.05) (Table 2).

Multiple regression analysis with the Stepwise method showed that employment status significantly predicted 6% of the variance of stress mean score and 11% of the variance of depression mean score (*p* < 0.05). Moreover, educational level and work shift significantly predicted 13% of the variance of anxiety mean score (*p* < 0.05) (Table 4).

**Table 4:** The results of multiple regression analysis to determine the predictors of depression, anxiety, and stress*

Problem	Predictors	R	R^2^	Adjusted R^2^	B	SE	Beta	T	P
Depression	Constant	0.344	0.149	0.114	10.016	0.684	–	14.647	0.001
	Employment status				1.453	0.267	0.344	5.442	0.001
Anxiety	Constant	0.386	0.118	0.138	1.965	2.773	–	0.709	0.479
	Educational level				0.173	0.063	0.182	2.734	0.007
	Work shift				0.021	0.009	0.136	2.171	0.031
Stress	Constant	0.261	0.068	0.060	8.326	2.388	–	3.487	0.001
	Employment status				0.118	0.057	0.141	2.071	0.039

*Depression Anxiety Stress Scale (DASS-21)[35]

## Discussion

This study assessed depression, anxiety, and stress among Iranian nurses who provided care to patients with COVID-19. The mean score and the prevalence rate of stress were 15.13 ± 4.76 and 54.9%, respectively. This is in line with the findings of several previous studies which reported that the prevalence of stress was 48% among nurses in Iran during the COVID-19 pandemic [43], 56% among nurses in Singapore during the Severe Acute Respiratory Syndrome epidemic [25], and 55.9% among healthcare providers in Egypt and Saudi Arabia during the COVID-19 pandemic [44]. These findings imply that more than half of the nurses who provide care to patients with COVID-19 suffer from stress. Stress can negatively affect their health and care quality [15, 30]. Therefore, strategies such as education about stress management strategies and psychological counseling and support are needed to reduce nurses’ stress.

The mean score and the prevalence rate of anxiety in the present study were respectively 13.21 ± 4.90 and 89.7%, while 73% of participants reported moderate to severe anxiety. This is in agreement with the findings of a study in Ethiopia which reported high levels of anxiety among nurses in COVID-19 care wards mostly due to unavailability of a guideline, fear of infecting family, and having chronic diseases. The prevalence of anxiety in that study was as high as 69.6% [31]. Several other studies also reported high levels of stress and anxiety among nurses in COVID-19 care wards mostly due to the necessity of long-term quarantine, fear over affliction by the disease, financial strains, despair, limited perceived support, and shortage of medications, equipment, and resources [16, 32, 45, 46]. However, anxiety prevalence in the present study was much higher than the anxiety prevalence rates in previous studies. For example, a study on 1257 healthcare providers in 34 hospitals in China reported an anxiety prevalence of 44.6% [29]. Moreover, a study during the COVID-19 pandemic found that the prevalence of anxiety among nurses in Iran was 38.8% [47]. A systematic review and meta-analysis on 93 studies also showed that the overall prevalence of anxiety among the 93,112 studied nurses was 37% [48]. Although the prevalence of anxiety among healthcare providers generally increases during infectious disease epidemics, the much higher anxiety prevalence in the present study compared with previous studies may be due to the differences among studies regarding contextual factors such as financial and sociocultural challenges.

Study findings also showed that the mean score and the prevalence rate of depression were respectively 13.56 ± 5.37 and 74.1%, and 43.7% of participants suffered from moderate to severe depression. The prevalence of depression among healthcare providers in studies conducted during the COVID-19 pandemic was 37.4%–64.7% [29, 43, 47, 49]. Depression can disturb thinking and functioning [43, 49]. The high prevalence of depression among healthcare providers highlights the necessity of healthcare authorities’ urgent attention and serious interventions to reduce mental health problems.

We also found that the mean scores of depression, anxiety, and stress had no significant relationship with age, gender, affiliated ward, work experience, and overtime work hours per month. In line with this finding, a study reported the insignificant relationship of age with depression, anxiety, and stress [50]. A study also found that the relationship of the mean scores of depression and stress with age, marital status, educational level, and work experience was not significant, while the mean scores of depression and anxiety among female nurses were more than male nurses [47]. Another study also reported higher anxiety and stress mean scores among female nurses [51]. Moreover, a study during the COVID-19 pandemic found higher depression among female physicians, higher stress among younger physicians, and higher anxiety among physicians with more weekly work hours [49]. The insignificant relationship of age and gender with the mean scores of depression, anxiety, and stress in the present study highlights the importance of careful attention to nurses of both genders and different age groups.

Our findings also showed that the mean scores of depression, anxiety, and stress had significant differences with employment status and were significantly higher among nurses who were doing their mandatory post-graduation services. This finding may be due to lower work experience, greater job insecurity, and higher workload of these nurses. Studies show that in the COVID-19 pandemic, nurses with lower work experience suffer from high levels of psychological strain due to their heavy workload and care provision to critically-ill patients [9, 51, 52]. Meanwhile, most nurses have limited access to mental healthcare services due to their heavy workload. Unstable employment status and limited job security also increase their stress and anxiety [9, 53].

Moreover, we found that the mean score of anxiety among nurses with master’s degree was significantly lower than nurses with bachelor’s degree. However, a former study did not find any significant relationship between educational level and mental health problems among nurses [47]. This inconsistency highlights the importance of further studies in this area. Another finding of the present study was the higher mean score of anxiety among nurses who did night shifts compared with nurses who did day or rotating shifts. In agreement with this finding, a previous study reported that the mean scores of depression, anxiety, and stress among physicians who did night or rotating shifts were significantly higher [49]. This may be due to the negative effects of fatigue, poor rest and sleep, and staff shortage on mental health among healthcare providers who do night shift. Long work hours, inappropriate work schedule, and separation from family contribute to mental health problems among nurses [52–54]. Therefore, strategies such as improvement of nurses’ work schedule and reduction of their work hours are needed to improve their mental health.

### Strengths

This study was conducted at national level and while nurses in Iran experienced high levels of psychological strain due to the shortage of medications, personal protective equipment, and vaccines approved by the World Health Organization resulting from extensive international sanctions against Iran.

### Limitations

One of the limitations of the present study was its cross-sectional uncontrolled design which provided no reliable data about causal relationships among the study variables. Moreover, we had no data about the mean scores of participants’ depression, anxiety, and scale before the COVID-19 pandemic. Data collection was performed online due to COVID-19-related restrictions. Moreover, data were collected through a single self-report instrument and hence, participants’ mental status might have affected their responses to the instruments. The instrument used in this study for mental health assessment is a valid instrument for the assessment of depression, anxiety, and stress but its data cannot be used for the diagnosis of mental health problems.

## Conclusion

This study suggests that most nurses who provide care to patients with COVID-19 suffer from depression, anxiety, and stress. Comprehensive interventions for the early diagnosis and management of the symptoms of depression, anxiety, and stress among nurses can prevent serious consequences, improve nursing care quality, and enhance patient satisfaction. Healthcare authorities and policy makers are recommended to employ serious interventions to identify nurses at risk for mental health problems and provide them with psychiatric counseling services as well as education about stress management in order to improve their mental health. The findings of this study can be used to develop plans for improving nurses’ employment status and reducing their work hours.

## Data Availability

Study data are accessible at formal request and with the permission of the authorities of the University of Social Welfare and Rehabilitation Sciences, Tehran, Iran.

## Abbreviations

COVID-19: Coronavirus disease 2019
DASS: Depression anxiety stress scales
STROBE: Strengthening the reporting of observational studies in epidemiology
SPSS: Statistical product and service solutions

## References

[1] De Angelis E, Renzetti S, Volta M, Donato F, Calza S, Placidi D, Lucchini RG, Rota M (2021). COVID-19 incidence and mortality in Lombardy, Italy: an ecological study on the role of air pollution, meteorological factors, demographic and socioeconomic variables. Environ Res.

[2] Worldometers. COVID-19 coronavirus pandemic. 2022. Available from: https://www.worldometers.info/coronavirus/

[3] Ahorsu DK, Lin CY, Imani V, Saffari M, Griffiths MD, Pakpour AH (2020). The fear of COVID-19 scale: development and initial validation. Int J Ment Health Addict.

[4] Moradi M, Navab E, Sharifi F, Namadi B, Rahimidoost M (2021). The Effects of the COVID-19 Pandemic on the Elderly: A Systematic Review. Salmand Iranian J Age.

[5] Ouyang L, Yu M, Zhu Y, Gong J (2021). Respiratory supports of COVID-19 patients in intensive care unit: a systematic review. Heliyon.

[6] Asadi N, Memarian R, Vanaki Z (2019). Motivation to care: a qualitative study on Iranian nurses. J Nurs Res.

[7] Gadecka W, Piskorz-Ogórek K, Regin KJ, Kowalski IM (2015). Social competence of mental health nurses. Polish Ann Med.

[8] Fernandez R, Lord H, Halcomb E, Moxham L, Middleton R, Alananzeh I (2020). Implications for COVID 19: a systematic review of nurses' experiences of working in acute care hospital settings during a respiratory pandemic. Int J Nurs Stud.

[9] Amiri A, Rashnuodi P, Mousavi SM, Shadian KL (2021). Investigating the level of job stress in nurses exposed to COVID-19 in educational hospitals in Ahvaz. J Occup Hyg Eng.

[10] Di Tella M, Benfante A, Castelli L, Romeo A (2021). Anxiety, depression, and post traumatic stress in nurses during the COVID-19 outbreak. Intensive Crit Care Nurs.

[11] Şanlıtürk D (2021). Perceived and sources of occupational stress in intensive care nurses during the COVID-19 pandemic. Intensive Crit Care Nurs.

[12] Liao KL, Huang YT, Kuo SH, Lin WT, Chou FH, Chou PL (2019). Registered nurses are at increased risk of hospitalization for infectious diseases and perinatal complications: A population-based observational study. Int J Nurs Stud.

[13] Pappa S, Ntella V, Giannakas T, Giannakoulis VG, Papoutsi E, Katsaounou P. Prevalence of depression, anxiety, and insomnia among healthcare workers during the COVID-19 pandemic: A systematic review and meta-analysis. Brain Behav Immun. 2020;88:901–907. doi: 10.1016/j.bbi.2020.05.026. Epub 2020 May 8. Erratum in: Brain Behav Immun. 2021 ;92:247 10.1016/j.bbi.2020.05.026 PMC7206431 32437915

[14] Alnazly E, Khraisat OM, Al-Bashaireh AM, Bryant CL (2021). Anxiety, depression, stress, fear and social support during COVID-19 pandemic among Jordanian healthcare workers. PLoS ONE.

[15] Asadi N, Salmani F, Pourkhajooyi S, Mahdavifar M, Rooyani Z, Mahin S (2020). Investigatingthe relationship between corona anxiety and nursing care behaviors working in coronary referral hospitals. Iranian J Psychiatry Clin Psychol.

[16] Garosi E, Khosravi M, Mazloumi A. Nurses and COVID-19 phenomenon: challenges and consequences. Iran Occupational Health. 2020;17: Special Issue: Covid-19.

[17] Pooladi M, Entezari M, Hashemi M, Bahonar A, Hushmandi K, Raei M (2020). Investigating the efficient management of different countries in the COVID-19 pandemic. J Mar Med.

[18] Rassouli M, Ashrafizadeh H, Shirinabadi Farahani A, Akbari ME (2020). COVID-19 Management in Iran as one of the most affected countries in the world: advantages and weaknesses. Front Public Health.

[19] McEwen BS. Stress, Definitions and Concepts of. Encyclopedia of Stress. 2010; 653. 10.1016/B978-012373947-6.00364-0

[20] Richardson KM, Rothstein HR (2008). Effects of occupational stress management intervention programs: a meta-analysis. J Occup Health Psychol.

[21] Veda A, Roy R (2020). Occupational stress among nurses: a factorial study with special reference to Indore city. J Health Manag.

[22] Crocq M-A (2015). A history of anxiety: from Hippocrates to DSM. Dialogues Clin Neurosci.

[23] Cohen SD, Cukor D, Kimmel PL (2016). Anxiety in patients treated with hemodialysis. Clin J Am Soc Nephrol..

[24] American Psychiatric Association. Diagnostic and Statistical Manual of Mental Disorders (DSM-5), Fifth edition. 2013.

[25] Koh D, Lim MK, Chia SE, Ko SM, Qian F, Ng V (2005). Risk perception and impact of severe acute respiratory syndrome (SARS) on work and personal lives of healthcare workers in Singapore what can we learn?. Med Care.

[26] Chen WK, Cheng YC, Chung YT, Lin CC (2005). The impact of the SARS outbreak on an urban emergency department in Taiwan. Med Care.

[27] Chua SE, Cheung V, Cheung C, McAlonan GM, Wong JW, Cheung EP, Chan MT, Wong MM, Tang SW, Choy KM, Wong MK, Chu CM, Tsang KW (2004). Psychological effects of the SARS outbreak in Hong Kong on high-risk health care workers. Can J Psychiatry.

[28] Khalid I, Khalid TJ, Qabajah MR, Barnard AG, Qushmaq IA (2016). Healthcare workers emotions, perceived stressors and coping strategies during a MERS-CoV outbreak. Clin Med Res.

[29] Lai J, Ma S, Wang Y, Cai Z, Hu J, Wei N (2020). Factors associated with mental health outcomes among health care workers exposed to coronavirus disease 2019. JAMA Network Open.

[30] Poursadeghiyan M, Abbasi M, Mehri A, Hami M, Raei M, Ebrahimi MH (2016). Relationship between job stress and anxiety, depression and job satisfaction in nurses in Iran. The Social Sciences.

[31] Mekonen E, Shetie B, Muluneh N (2021). The psychological impact of COVID-19 outbreak on nurses working in the Northwest of Amhara regional state referral hospitals. Northwest Ethiopia Psychol Res Behav Manag.

[32] Khaki S, Fallahi-Khoshkenab M, Arsalani N, Mojtaba R, Sadeghy N, Nematifard T (2021). Mental health status of nurses during the COVID-19 pandemic: a systematic review. Mental Health.

[33] Vandenbroucke JP, von Elm E, Altman DG, Gotzsche PC, Mulrow CD, Pocock SJ, Poole C, Schlesselman JJ, Egger M (2007). Strengthening the reporting of observational studies in epidemiology (STROBE): explanation and elaboration. Epidemiology.

[34] Ebadi A, Zarshenas L, Rakhshan M, Zareian A, Sharifnia H, Mojahedi M (2017). Principles of scale development in health science.

[35] Antony MM, Bieling PJ, Cox BJ, Enns MW, Swinson RP (1998). Psychometric properties of the 42-item and 21-item versions of the depression anxiety stress scales in clinical groups and a community sample. Psychol Assess.

[36] Lovibond SH, Lovibond PF (1995). Manual for the depression anxiety stress scales.

[37] Sinclair SJ, Siefert CJ, Slavin-Mulford JM, Stein MB, Renna M, Blais MA (2012). Psychometric evaluation and normative data for the depression, anxiety, and stress scales-21 (DASS-21) in a nonclinical sample of US adults. Eval Health Prof.

[38] Harris M, Wilson JC, Holmes S, Radford DR (2017). Perceived stress and well-being among dental hygiene and dental therapy students. Br Dent J.

[39] Sahebi A, Asghari MJ, Salari RS (2005). Validation of depression anxiety and stress scale (DASS-21) for an Iranian population. J Iranian Psychol.

[40] Samani S, Jokar B (2007). Evaluation of the validity of the short scale of depression, anxiety and stress. Shiraz Univ J Soc Sci Humanit.

[41] Henry JD, Crawford JR (2005). The short-form version of the depression anxiety stress scales (DASS-21): construct validity and normative data in a large non-clinical sample. Br J Clin Psychol.

[42] Asghari A, Saed F, Dibajnia P (2008). Psychometric properties of the depression anxiety stress scales-21 (DASS-21) in a non-clinical Iranian sample. Int J Psychol.

[43] Sarboozi Hosein Abadi T, Askari M, Miri K, Nia MN (2020). Depression, stress and anxiety of nurses in COVID-19 pandemic in Nohe-Dey Hospital in Torbat-e-Heydariyeh city, Iran. J Mil Med.

[44] Arafa A, Mohammed Z, Mahmoud O, Elshazley M, Ewis A (2020). Depressed, anxious, and stressed: What have healthcare workers on the frontlines in Egypt and Saudi Arabia experienced during the COVID-19 pandemic?. J Affect Disord.

[45] Liu C-Y, Yang Y-z, Zhang X-M, Xu X, Dou QL, Zhang W-W (2020). The prevalence and influencing factors in anxiety in medical workers fighting COVID-19 in China: a cross-sectional survey. Epidemiol Infect.

[46] Grimm CA. Hospital Experiences Responding to the COVID-19 Pandemic: Results of a National Pulse Survey March 23–27, 2020. OEI-06-20-00300.

[47] Pouralizadeh M, Bostani Z, Maroufizadeh S, Ghanbari A, Khoshbakht M, Alavi SA (2020). Anxiety and depression and the related factors in nurses of Guilan university of medical sciences hospitals during COVID-19: a web-based cross-sectional study. Int J Africa Nurs Sci.

[48] Al Maqbali M, Al Sinani M, Al-Lenjawi B (2021). Prevalence of stress, depression, anxiety and sleep disturbance among nurses during the COVID-19 pandemic: a systematic review and meta-analysis. J Psychosom Res.

[49] Elbay RY, Kurtulmuş A, Arpacıoğlu S, Karadere E (2020). Depression, anxiety, stress levels of physicians and associated factors in Covid-19 pandemics. Psychiatry Res.

[50] Zheng R, Zhou Y, Qiu M, Yan Y, Yue J, Yu L, Lei X, Tu D, Hu Y (2021). Prevalence and associated factors of depression, anxiety, and stress among Hubei pediatric nurses during COVID-19 pandemic. Compr Psychiatry.

[51] Dai Y, Hu G, Xiong H, Qiu H, Yuan X (2020). Psychological impact of the coronavirus disease 2019 (COVID 19) outbreak on healthcare workers in China. MedRxiv.

[52] Zamanzadeh V, Valizadeh L, Khajehgoodari M (2021). Nurses’ experiences during the COVID-19 pandemic in Iran: a qualitative study. BMC Nurs.

[53] Sharifi Fard F, Nazari N, Asayesh H, Ghanbari Afra L, Goudarzi Rad M, Ghodrati M (2021). Evaluationof psychological disorders in nurses facing infected Covid-19 patients in 2020. Qom Univ Med Sci J.

[54] Karimi Z, Fereidouni Z, Behnammoghadam M, Alimohammadi N, Mousavizadeh A, Salehi T, Mirzaee MS, Mirzaee S (2020). The lived experience of nurses caring for patients with COVID-19 in Iran: a phenomenological study. Risk Manag Healthc Policy.

